# Application of VVI layered strain technology to analyze changes in left ventricular myocardial motion in patients with uremia

**DOI:** 10.1371/journal.pone.0336766

**Published:** 2026-03-20

**Authors:** Xinyan Dai, Yihua Gao, Xianglan Fang, Hailan Zheng

**Affiliations:** 1 Department of Medical Ultrasound, Yanbian University Hospital, Yanji, Yanbian, China; 2 Department of Nephrology, Yanbian University Hospital, Yanji, Yanbian, China; CUNY School of Medicine: The City College of New York CUNY School of Medicine, UNITED STATES OF AMERICA

## Abstract

**Objective:**

Velocity vector imaging (VVI) stratified strain analysis was used to assess circumferential strain across myocardial layers in uremic patients using a 16-segment left ventricular model.

**Methods:**

Data were accessed for research purposes from 1/5/2025–1/6/2025. Sixty hemodialysis patients were divided into left ventricular hypertrophy (LVH, n = 30) and normal LV conformation (LVN, n = 30) groups based on LV mass index, alongside 27 healthy controls (CON). Clinical and echocardiographic data were analyzed, with VVI quantifying systolic circumferential strain (CS) in endocardial (Endo), mid-myocardial (Myo), and epicardial (Epi) layers.

**Results:**

The LVH and LVN groups exhibited significant differences compared to the CON group in SBP, Hb, BUN, Scr, eGFR, and hsCRP levels (*P* < 0.001). Hb levels were uniquely reduced in LVH versus LVN (*P* < 0.05). Both uremic groups had larger cardiac dimensions (LAD, LVPTW, IVST, LVIDd, LVIDs) than CON (*P* < 0.001), but similar LVEF (*P* > 0.05). Basal and mid-segment global strain were impaired in LVH/LVN (*P* < 0.05), while apical strain differences were only significant in the Epi layer. LVH showed reduced anteroseptal basal strain (Endo/Myo/Epi-2CS) and inferoseptal basal (Endo/Myo-3CS) and anterolateral mid (Epi-12CS) strain versus LVN. Anteroseptal (Endo/Myo/Epi-2CS) and inferoseptal (Endo/Myo-3CS) strains correlated with Hb levels (*P* < 0.01). Anteroseptal basal strains (Endo/Myo/Epi-2CS) and their combined values significantly predicted myocardial remodeling (AUC, *P* < 0.001). While Epi-3CS and its combined values (Endo/Myo/Epi-2CS) did not (*P* > 0.05).

**Conclusions:**

The VVI layered strain technique is a precise and sensitive tool for evaluating LV myocardial motion in uremic patients.

## Introduction

Chronic kidney disease (CKD) is characterized by irreversible, progressive deterioration of kidney structure and function [1,2]. Its morbidity and mortality rates remain alarmingly high, with a growing burden across Asia and globally [3,4]. Notably, China and India account for the highest proportion of CKD cases in Asia, as evidenced by the Asian CKD Epidemiology Survey and corroborated by the Global Burden of Disease database [5]. The United States faces a substantial burden of end-stage renal disease (ESRD), characterized by one of the highest incidence rates globally, which continues to rise. Concurrently, the prevalence of ESRD has also shown a persistent upward trend [6]. Uremia, the terminal stage of CKD, arises from chronic renal failure and impairs cardiac morphology, structure, and function due to systemic toxin accumulation from impaired renal clearance [7,8]. Among CKD patients, a significant number of patients succumb to cardiovascular disease (CVD) even before progressing to ESRD [9], with LVH and congestive heart failure being the most prevalent underlying causes [10], particularly those with ESRD—CVD mortality and morbidity exceed those of the general population [11]. Strikingly, over 50% of ESRD patients succumb to CVD, underscoring its clinical significance [12]. A subset of ESRD patients with superimposed acute kidney injury may experience renal recovery sufficient to discontinue dialysis, although ESRD is itself defined by irreversible renal loss [13]. Furthermore, hemodialysis has been shown in numerous studies to alleviate left ventricular hypertrophy and reverse systolic dysfunction in this population [14,15]. Early detection, diagnosis, and intervention for CVD are thus critical to improving survival rates and quality of life in ESRD patients. Timely management may mitigate CVD-related risks in this high-risk cohort, offering substantial benefits to long-term health outcomes.

Velocity vector imaging (VVI), based on two-dimensional speckle tracking echocardiography (2D-STE), represents an advanced echocardiographic technique for noninvasive cardiac function assessment. Unlike conventional echocardiography, VVI is independent of Doppler angle limitations, demonstrating superior reproducibility, objectivity, and comprehensive evaluation of myocardial motion and global cardiac function [16,17]. Previous studies indicate that the left ventricular basal segment is particularly susceptible to impairment during myocardial stress in uremic patients. Accordingly, this study employed the VVI layered strain technique to analyze systolic circumferential strain (CS) across three myocardial layers and six segments of the left ventricular basal segment. We compared measurements among three groups: uremic patients with normal left ventricular geometry, those with left ventricular hypertrophy, and healthy controls. Our findings provide valuable insights into the clinical utility of VVI for assessing myocardial function in uremia.

## Materials and methods

### Study population

We enrolled 60 uremic patients undergoing maintenance hemodialysis (≥ 3sessions/week for ≥ 6months) at our nephrology department from June 2023 to June 2024. Diagnosis followed the 2012 K/DOQI guidelines (GFR < 10mL/min and serum creatinine > 707μmol/L, equivalent to stage 5 CKD). Exclusion criteria included: LVEF < 55%, segmental wall motion abnormalities, arrhythmias, significant valvular disease, or congenital heart disease. Data inclusion was limited to echocardiograms acquired on the second interdialytic day to minimize the influence of acute volume shifts on cardiac structural and functional measurements. This retrospective clinical study was approved by the Ethics Committee of Yanbian University Hospital (2025231), and the Ethics Committee waived informed consent.The authors did not have access to information that could identify individual participants during or after data collection.

Participants were stratified by left ventricular mass index (LVMI) into: Normal left ventricular geometry (LVN group; n = 30), LVMI < 115g/m^2^ (men) or <95g/m^2^ (women); Left ventricular hypertrophy (LVH group; n = 30), LVMI ≥ 115g/m^2^ (men) or ≥ 95g/m^2^ (women). A control group (n = 27) of age-, gender-, and BMI-matched healthy individuals undergoing routine health examinations during the same period was included for comparison.

### Instrument and method

Echocardiography was performed using a Siemens SC2000 system (4V1 probe, 1–4MHz) with VVI software. Two-dimensional echocardiography with three or more consecutive cardiac cycles and dynamic image acquisition was performed by a senior physician on the three groups of patients. Left ventricular mass was measured using the linear 2D method as recommended by the American Society of Echocardiography. Left atrium diameter (LAD), left ventricular posterior wall thickness (LVPTW), interventricular septal thickness (IVST), left ventricular end-diastolic and end-systolic internal diameter (LVIDd and LVIDs), and left ventricular ejection fraction (LVEF), as well as basic information such as the patient’s age, gender, BMI, systolic blood pressure (SBP), diastolic blood pressure (DBP), hemoglobin (Hb), blood urea nitrogen (BUN), serum creatinine (Scr), estimated glomerular filtration rate (eGFR), and hypersensitive C-reactive protein (hsCRP), were recorded, and the LVMI was calculated according to the formula recommended by the American Society of Echocardiography (ASE) [18] as LVMI = Left ventricular mass (LVM)/Body surface area (BSA), LVM = 1.04× [(LVIDd+IVST+LVPTW)^3^-

**Fig 1:**
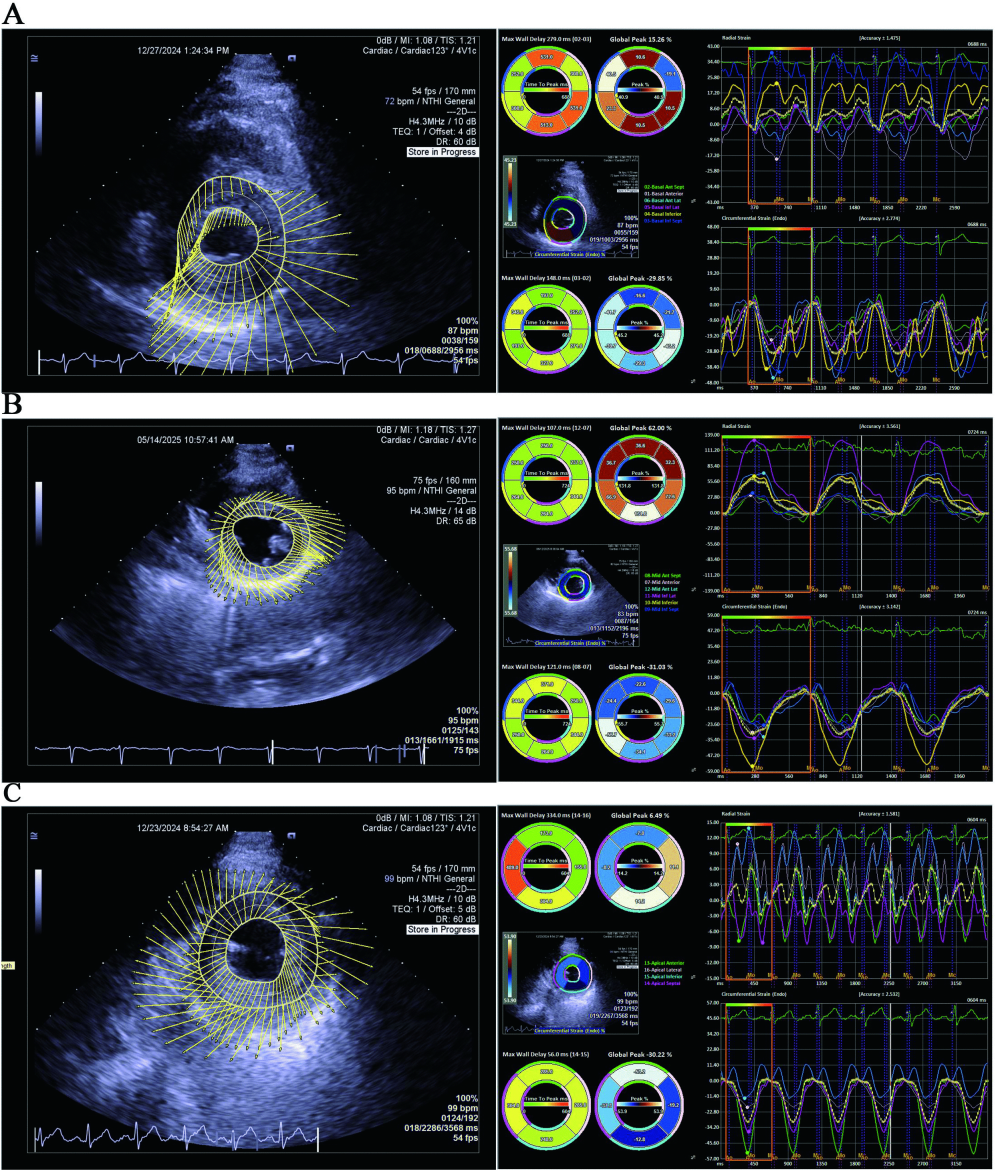
VVI technique for displaying myocardial circumferential motion parameters. A. Velocity vector imaging shows basal myocardial motion, and VVI software analyzes myocardial strain. B. Velocity vector imaging shows mid-myocardial motion, and VVI software analyzes myocardial strain. C. Velocity vector imaging shows apical myocardial motion, and VVI software analyzes myocardial strain.

LVIDd^3^]×0.8 + 0.6, BSA = 0.0061 × height(cm)+0.0128 × weight(kg)-0.1529. Endocardial border tracing was performed in three parasternal short-axis views using velocity vector imaging (VVI) software. The system automatically delineated the epicardial border and analyzed three distinct myocardial layers: endocardium (Endo), mid-myocardium (Myo), and epicardium (Epi), with manual adjustments made according to actual myocardial thickness. Circumferential strain data for all 16 segments across each myocardial layer are presented in Fig 1.

To ensure research transparency and reproducibility, the de-identified individual-level raw data for all participants in this study—including demographic information, laboratory test indicators, and left ventricular global and layer-specific strain parameters obtained via VVI technology—have been provided as a Supporting Information file (S1 Data).

### Statistical analysis

A post hoc power analysis was conducted using G*Power software (version 3.1.9.2) to evaluate the adequacy of the sample size. We performed an approximate calculation with the one-way analysis of variance (F-test) as the model and a moderate effect size (f = 0.35) set. The analysis showed that with our current sample size of 87, the statistical power reached 82% to detect a clinically significant effect, indicating that the current sample is adequate. Data analysis was performed using SPSS 25.0 (IBM Corp.), with GraphPad Prism 9.5 and MedCalc employed for graphical representations. Categorical variables were compared using the chi-square test. Normality was assessed by synthesizing the results of the Shapiro-Wilk test with visual inspection of quantile-quantile (Q-Q) plots. Normally distributed continuous variables are expressed as mean±SD and analyzed via one-way ANOVA. Non-normally distributed data are presented as median [25th-75th percentiles], with between-group comparisons conducted using the Kruskal-Wallis test (multiple groups) or Mann-Whitney U test (two groups). Correlation analyses utilized Pearson (normal distribution) or Spearman (non-normal distribution) coefficients. Receiver operating characteristic (ROC) curves evaluated the discriminatory power of VVI parameters, with specificity, sensitivity, and area under the curve (AUC) quantified. Statistical significance was defined as *P* < 0.05.

### Reproducibility analysis

To evaluate the reproducibility of VVI measurements, images from 15 randomly selected subjects were analyzed. Intra-observer reproducibility was assessed by having a senior sonographer (Reader A) re-measure all images after a 4-week interval. Inter-observer reproducibility was evaluated by an independent, blinded sonographer (Reader B) of comparable experience. Agreement was quantified using the intraclass correlation coefficient (ICC) based on a two-way random-effects model, with ICC values > 0.75 indicating good agreement.

## Results

### Comparison of basic clinical information

A comparison of the LVH and LVN groups with the CON group revealed statistically significant differences in the levels of SBP, Hb, BUN, Scr, eGFR, and hsCRP (*P* < 0.001). In contrast, no statistically significant differences were found regarding age, gender, BMI, and DBP (*P* > 0.05). Additionally, significant differences in Hb levels were observed between the LVH and LVN groups (*P* < 0.05). (Table 1)

**Table 1:** Comparison of basic clinical information on study participants.

Indicators	LVH（N = 30)	LVN (N = 30)	CON (N = 27)	F/χ^2^/H	*P*	η²/ε²/Cramér’s V	*95%CI (LVHvsLVN)*
**Age, SD**	65.43 ± 7.07	65.27 ± 5.46	64.85 ± 4.51	0.074	0.928	0.002	[-2.82, 3.15]
**Male, n(%)**	15(50.00)	15(50.00)	13(48.10)	0.026	0.987	0.017	–
**BMI(kg/m**^**2**^)	23.37 ± 3.27	23.20 ± 1.70	22.67 ± 1.70	0.912	0.408	0.016	[-1.50, 1.84]
**SBP(mmHg)**	150.10 ± 20.27^a^	145.83 ± 19.54^a^	123.33 ± 6.08	37.645	<0.001	0.325	[-8.37, 16.90]
**DBP(mmHg)**	79.50 ± 12.82	75.60 ± 10.78	79.56 ± 3.94	1.750	0.185	0.034	[-3.63, 11.43]
**Hb(g/L)**	88.37 ± 17.59^ab^	97.23 ± 17.05^a^	128.22 ± 5.17	104.417	<0.001	0.679	[-26.44, -6.22]
**BUN(mmol/L)**	20.85 ± 6.11^a^	18.82 ± 9.11^a^	4.85 ± 0.76	132.532	<0.001	0.547	[-2.91, 6.98]
**Scr(umol/L)**	557.00 [468.00,599.00]^a^	513.00 [199.00, 659.50]^a^	66.00 [60.00, 72.00]	55.523	<0.001	0.645	[-42.25, 127.85]
**eGFR(mL/ min/1.73m**^**2**^)	8.83 [6.26, 10.42]^a^	14.31 [6.85, 19.90]^a^	106.20 [100.50, 115.20]	56.667	<0.001	0.658	[-8.92, 2.96]
**hsCRP(mg/L)**	7.99 [6.55, 19.26]^a^	6.60 [2.20, 21.31]^a^	2.50 [0.94, 3.21]	24.568	<0.001	0.289	[-2.15, 4.63]

^a^ compared with CON group, *P* < 0.05; ^b^ compared with LVN group, *P* < 0.05.

SBP: systolic blood pressure; DBP: diastolic blood pressure; Hb: hemoglobin; BUN: blood urea nitrogen; Scr: serum creatinine; eGFR: estimated glomerular filtration rate; hsCRP: hypersensitive C-reactive protein.

### Comparison of conventional echocardiographic parameters

The comparison of LVH and LVN groups with the CON group revealed statistically significant differences in several conventional echocardiographic parameters, including LAD, LVPTW, IVST, LVIDd, and LVIDs (*P* < 0.001). However, the differences in LVEF between these groups were not statistically significant (*P* > 0.05). When comparing LVH and LVN directly, significant differences were noted for LVPTW, IVST, and LVIDd (*P* < 0.05). In contrast, no statistically significant differences were observed for LAD, LVIDs, and LVEF (*P* > 0.05). For more detailed results (Table 2).

**Table 2:** Comparison of conventional echocardiographic parameters in study subjects.

Indicators	LVH (N = 30)	LVN (N = 30)	CON (N = 27)	F	*P*	η²	*95%CI (LVHvsLVN)*
**LAD(mm)**	35.43 ± 5.55^a^	34.10 ± 2.90^a^	30.26 ± 3.59	12.718	<0.001	0.215	[-1.51, 4.17]
**LVPTW(mm)**	10.33 ± 0.99^ab^	8.80 ± 1.00^a^	7.85 ± 0.77	51.883	<0.001	0.553	[1.06, 2.01]
**IVST(mm)**	10.20 ± 1.03^ab^	8.80 ± 1.00^a^	7.89 ± 0.80	42.808	<0.001	0.505	[0.91, 1.89]
**LVIDd(mm)**	51.83 ± 3.09^ab^	49.70 ± 2.20^a^	44.59 ± 1.85	76.654	<0.001	0.606	[0.43, 3.84]
**LVIDs(mm)**	32.17 ± 3.41^a^	31.10 ± 3.68^a^	26.78 ± 1.69	38.193	<0.001	0.361	[-1.19, 3.32]
**LVEF(%)**	58.50 ± 1.72	58.77 ± 2.28	59.81 ± 2.65	3.077	0.215	0.060	[-1.05, 1.02]

^a^ compared with CON group, *P* < 0.05; ^b^ compared with LVN group, *P* < 0.05.

LAD: left atrium diameter; LVPTW: left ventricular posterior wall thickness; IVST: interventricular septal thickness; LVIDd: left ventricular end-diastolic internal diameter; LVIDs: left ventricular end-systolic internal diameter; LVEF: left ventricular ejection fraction.

### Comparison of VVI layered strain parameters

**Fig 2:**
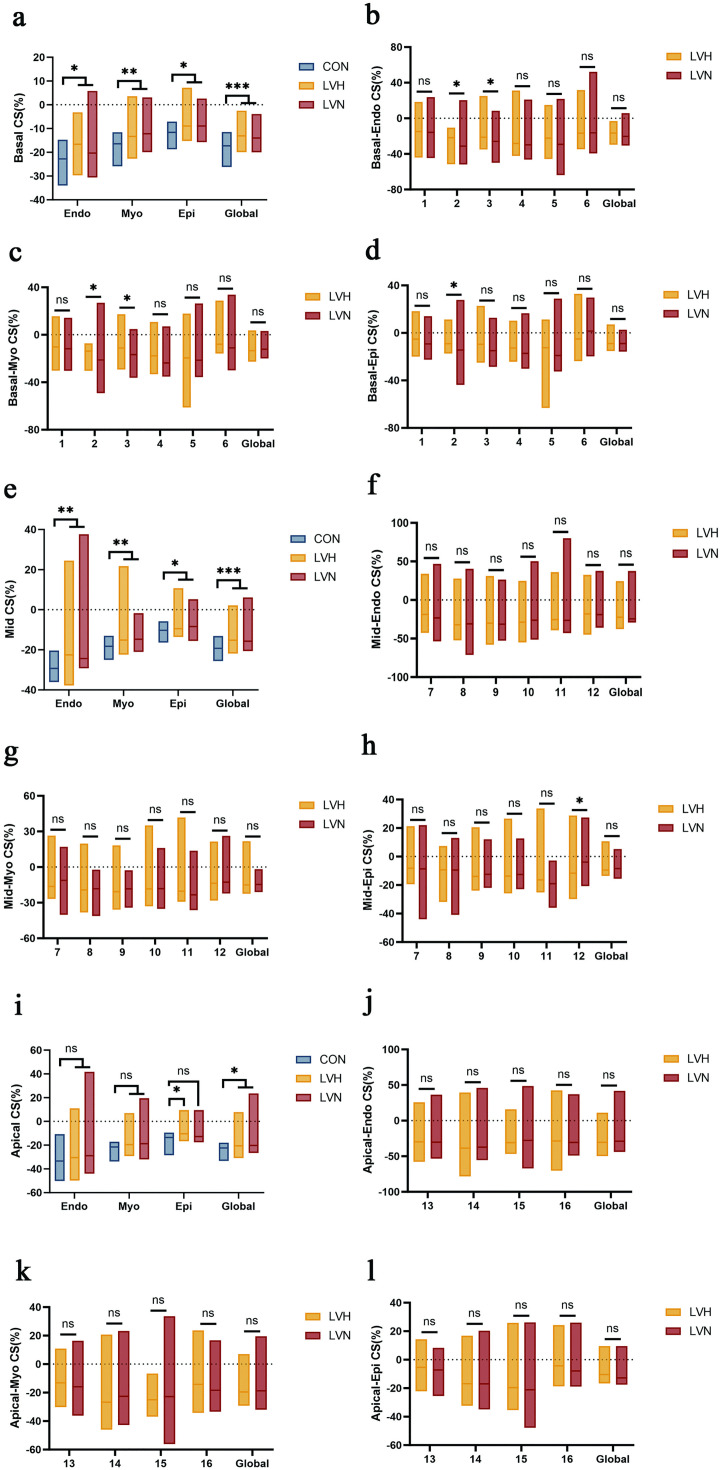
Comparison of VVI parameters in the study population. a. CON compared with the LVH and LVN groups in the circumferential strain in the basal segment in the Endo, Myo, Epi and the global strain in all three layers; b. Comparison of segments of the Endo in basal segments between the LVH and LVN groups; c. Comparison of segments of the Myo in basal segments between the LVH and LVN groups; d. Comparison of segments of the Epi in basal segments between the LVH and LVN groups; e. CON compared with the LVH and LVN groups in the circumferential strain in the mid segment in the Endo, Myo, Epi and the global strain in all three layers; f. Comparison of segments of the Endo in mid segments between the LVH and LVN groups; g. Comparison of segments of the Myo in mid segments between the LVH and LVN groups; h. Comparison of segments of the Epi in mid segments between the LVH and LVN groups; i. CON compared with the LVH and LVN groups in the circumferential strain in the apical segment in the Endo, Myo, Epi and the global strain in all three layers; j. Comparison of segments of the Endo in apical segments between the LVH and LVN groups; k. Comparison of segments of the Myo in apical segments between the LVH and LVN groups; l. Comparison of segments of the Epi in apical segments between the LVH and LVN groups.

Comparative analysis revealed significant differences in circumferential strain parameters between the CON group and both LVH and LVN groups in basal and Mid myocardium (*P* < 0.05). In the comparison of circumferential strain at the apex, CON showed statistical differences from LVH in Epi(*P* < 0.05). At the Global level, CON showed statistical differences from both LVH and LVN (*P* < 0.05). Whereas there was a statistical difference in the 2CS between LVH and LVN in the comparison between the layers of the Endo, Myo, and Epi (*P* < 0.05), and in the Endo and Myo, in which 3CS was statistically different (*P* < 0.05), in the Epi, 12CS showed statistical differences (*P* < 0.05) (Fig 2).

### Comparison of VVI parameter correlations in total populations

**Fig 3:**
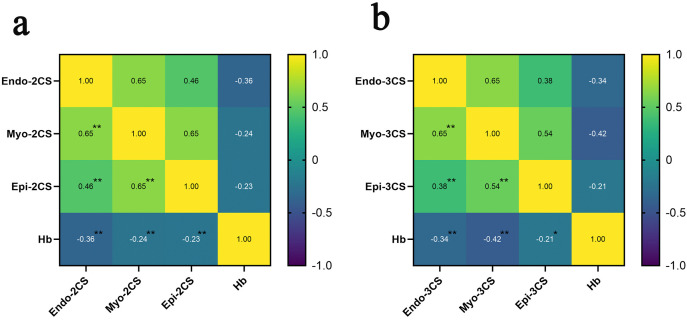
Comparison of VVI parameter correlations in total populations. a. Correlation analysis of Endo, Myo and Epi myocardium and Hb within the Anteroseptal Basal; b. Correlation analysis of Endo, Myo and Epi myocardium and Hb within the Inferoseptal Basal. Note: **P < 0.01.

There was a correlation between Endo-2CS, Myo-2CS, Epi-2CS and Hb levels (*P* < 0.01); and between Endo-3CS, Myo-3CS, Epi-3CS and Hb levels (*P* < 0.01) (Fig 3).

### ROC analysis of myocardial remodeling in uremia by VVI values

**Fig 4:**
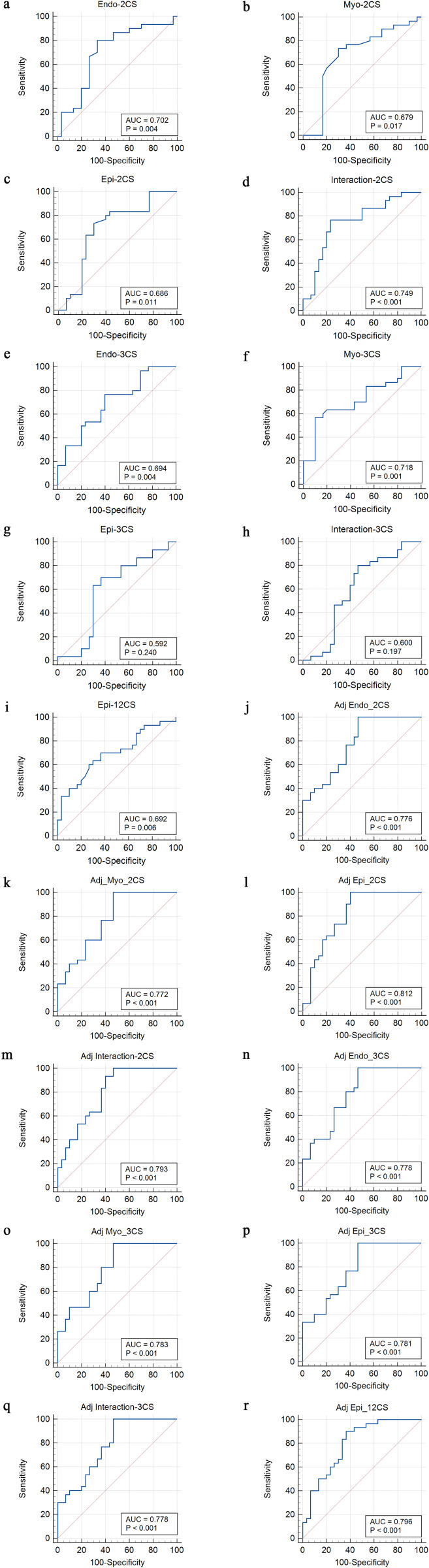
ROC analysis of myocardial remodeling in uremia by VVI values. The levels of Endo-2CS, Myo-2CS, Epi-2CS, Interaction-2CS and Epi-12CS were statistically significant in identifying the AUC of myocardial remodeling in uremic patients (a,b,c,d, i). The levels of Endo-3CS and Myo-3CS were statistically significant in identifying the AUC of myocardial remodeling in uremic patients (e,f). There was no statistical significance in the levels of Epi-3CS and Interaction-3CS (g,h). After adjusting for the influencing factor of Hb, the levels of Endo-2CS,Myo-2CS, Epi-2CS, Interaction-2CS, Endo-3CS, Myo-3CS, Epi-3CS, Interaction-2CS, Interaction-3CS and Epi-12CS were statistically significant in identifying the AUC of myocardial remodeling in uremic patients (j,k,l,m,n,o,p,q,r).

In ROC analysis, Endo-2CS, Myo-2CS, Epi-2CS, Interaction-2CS, and Epi-12CS were statistically significant (*P* < 0.05) in identifying the AUC of myocardial remodeling in uremic patients, and the levels of Endo-3CS and Myo-3CS were statistically significant (*P* < 0.05) in identifying the AUC of myocardial remodeling in uremic patients. However, there was no statistical significance (*P* > 0.05) in the levels of Epi-3CS and Interaction-3CS. After adjusting for the influencing factor of Hb, Endo-2CS, Myo-2CS, Epi-2CS, Interaction-2CS, Endo-3CS, Myo-3CS, Epi-3CS, Interaction-3CS, Epi-12CS were statistically significant (*P* < 0.05) in identifying the AUC of myocardial remodeling in uremic patients (Table 3, Fig 4).

**Table 3:** ROC analysis of myocardial remodeling in uremia by VVI values.

Parameters	sensitivity	specificity	threshold	AUC	AUC_adj_	*P*	*P*_*adj*_
**Endo-2CS**	80.0	66.7	>-26.6	0.702	0.776	0.004	0.001
**Myo-2CS**	76.7	70.0	>-16.3	0.679	0.772	0.017	0.001
**Epi-2CS**	76.7	76.7	>-10.6	0.686	0.812	0.011	0.001
**Interaction-2CS**	76.7	80.0	–	0.749	0.793	0.001	0.001
**Endo-3CS**	73.3	63.3	>-25.3	0.694	0.778	0.004	0.001
**Myo-3CS**	56.7	90.0	>-12.2	0.718	0.783	0.001	0.001
**Epi-3CS**	70.0	56.7	>-14.6	0.592	0.781	0.240	0.001
**Interaction-3CS**	80.0	53.3	–	0.600	0.778	0.197	0.001
**Epi-12CS**	60.0	73.3	>-11.2	0.692	0.796	0.006	0.001

Endo-2CS: circumferential strain of endocardial anteroseptal basal; Myo-2CS: circumferential strain of myocardial anteroseptal basal; Epi-2CS: circumferential strain of epicardial anteroseptal basal; Interaction-2CS: the combined index (Endo-2CS × Myo-2CS × Epi-2CS); Endo-3CS: circumferential strain of endocardial inferoseptal basal; Myo-3CS: circumferential strain of myocardial inferoseptal basal; Epi-3CS: circumferential strain of epicardial inferoseptal basal; Interaction-3CS: the combined index (Endo-3CS × Myo-3CS × Epi-3CS); Epi-12CS: circumferential strain of epicardial anterolateral mid. adj: Adjusted the influencing factors of Hb.

### Reproducibility analysis results

The reproducibility analysis for GCS demonstrated excellent intra-observer and inter-observer agreement. Specifically, the ICCs for GCS at different left ventricular short-axis levels were as follows: for basal-segment GCS, the intra-observer ICC was 0.95 and the inter-observer ICC was 0.94; for mid-segment GCS, the intra-observer ICC was 0.98 and the inter-observer ICC was 0.98; and for apical-segment GCS, the intra-observer ICC was 0.99 and the inter-observer ICC was 0.88. Detailed data are presented in Table 4.

**Table 4:** Reliability analysis of the Basal- and Mid- and Apical-GCS(n = 15).

Parameters	Intraobserver ICC (95% CI)	Interobserver ICC (95% CI)
**Basal-GCS**	0.95 (0.84-0.98)	0.94 (0.84-0.98)
**Mid-GCS**	0.98 (0.96-0.99)	0.98 (0.93-0.99)
**Apical-GCS**	0.99 (0.99-0.99)	0.88 (0.65-0.96)

## Discussion

CVD accounts for over 50% of mortality in patients with ESRD. This elevated risk is primarily attributed to cardiovascular alterations induced by renal insufficiency [19]. Patients with ESRD frequently present with cardiovascular abnormalities, including both systolic and diastolic dysfunction, as well as pronounced left ventricular hypertrophy [20]. Given these complications, reliable and noninvasive methods are crucial for early detection of myocardial structural and functional impairments in this population. While LVEF and ventricular wall motion analysis via echocardiography remain standard clinical tools for assessing left ventricular systolic function, LVEF often remains normal in early-stage disease [21]. Notably, CKD patients with reduced LVEF face increased morbidity and mortality risks. Intriguingly, those with preserved LVEF but concomitant diastolic dysfunction exhibit even worse outcomes compared to patients with impaired systolic function [22]. The VVI layered strain technique, based on 2D-STE, quantifies myocardial motion direction and velocity through vector analysis, thereby overcoming several limitations of conventional echocardiography. Badran et al. [23] demonstrated the feasibility and reproducibility of VVI as a novel tracking method, showing its accuracy in evaluating left ventricular longitudinal mechanics in complex conditions such as hypertrophic cardiomyopathy (HCM), with performance comparable to three-dimensional speckle-tracking echocardiography (3D-STE). However, while 2D-STE cannot comprehensively assess myocardial layer-specific function, 3D-STE—though superior in evaluating left ventricular dysfunction [24–26]—suffers from lower spatiotemporal resolution and stringent image quality requirements, limiting its routine clinical application [27]. Zhu et al. used VVI in post-percutaneous coronary intervention (PCI) patients, showing elevated peak longitudinal and radial strain after one month and demonstrating its utility in monitoring myocardial reperfusion [28]. Zhang et al. applied VVI to differentiate primary light-chain cardiac amyloidosis (AL-CA) from HCM, identifying a characteristically severe reduction in endocardial longitudinal strain in AL-CA patients, thereby establishing it as a sensitive indicator for this differential diagnosis [29]. Wang et al. employed VVI to analyze left ventricular systolic function in uremic dialysis patients, reporting that longitudinal peak strain sensitivity was highest in the basal segment post-dialysis, followed by the intermediate and apical segments [30]. This suggests that hemodialysis predominantly impacts the basal and mid-ventricular regions, which endure greater mechanical stress due to their stronger contractile properties. Similarly, Ma et al. used 2D-STE to identify subclinical myocardial dysfunction in uremic patients with preserved LVEF, observing reduced longitudinal strain and prolonged peak strain time in the basal segment compared to healthy controls [31]. Circumferential myocardial fiber deformation demonstrates superior ultrasound detectability compared to longitudinal fiber deformation, owing to its relative insensitivity to global cardiac motion artifacts. These studies from diverse disease contexts collectively underscore the significant potential of VVI layered strain technology in quantifying myocardial motion and precisely assessing specific pathophysiological changes. Building on these findings, our study applies the VVI layered strain technique to assess myocardial motion in short-axis mitral valve basal views in uremic patients with normal LVEF.

The pathogenesis of uremic cardiomyopathy is complex and multifactorial, and common etiologic factors include left ventricular pressure and volume overload, disturbances in electrolyte metabolism, inflammation, and enhanced oxidative stress [20,32]. In this study, the LAD, LVPTW, IVST, LVIDd and LVIDs of LVH and LVN groups were higher than those of CON group, which may be attributed to the fact that in the early stage of uremia when the cardiac structure is not mutated, the weakening of renal water and sodium metabolism leads to water and sodium retention and increase in blood volume, and on the one hand, the heart can compensate for this increase in volume load by increasing its contractile capacity, and on the other hand, it can stimulate the renin-angiotensin- aldosterone system (RAS system) activation to compensate for the increase in volume load, so that the left ventricular contractility is maintained in the normal range, but as the disease reaches the advanced stage, the compensatory mechanism gradually fails, making the heart enlarged and myocardial hypertrophy. Although LVEF was normal in both the LVH and LVN groups, the elevation of the above left heart function parameters indicated that cardiac function was already impaired in patients in the LVH and LVN groups.

In this study, the overall strain of each layer of the 16 segments myocardium in each group was analyzed and it was concluded that there were significant differences in the level of Endo, Myo, Epi, and Global strains in the CON group compared with the LVH and LVN groups in basal and mid myocardium, but there was no significant difference between the LVH and LVN groups, suggesting that in the level of overall strain in each layer and in the whole layer the LVH and LVN groups behaved similarly, but were significantly different from the CON group, which suggests that in the LVEF is normal the myocardium of uremic patients has also been damaged. Although there was no difference between the LVH and LVN groups in terms of overall strain levels, the present study further analyzed the strain of the two groups in each segment at each level to conclude that among the three layers of myocardial strain in the LV (after adjusting for the influencing factor of Hb), there was a significant difference in the level of myocardial strain in the basal anteroseptal and inferoseptal segments, and there was a significant difference in the anterolateral mid in the Epi layer. There was a significant difference between the two groups in the all layers of basal inferoseptal, suggesting that the segments more prone to injury in patients with uremic left ventricular hypertrophy are the basal anteroseptal, basal inferoseptal and the Epi layer of mid-anterolateral segment, which may be since Basal Anteroseptal is located in the anterior aspect of the left ventricle between the anterior papillary muscle and anterior septum. The basal anteroseptal and basal inferoseptal segments primarily receive blood supply from the left anterior descending artery (LAD), while the mid-anterolateral segment is mainly perfused by the left circumflex artery (LCX). As the two major branches of the left coronary artery (LCA), both the LAD and LCX are critical for myocardial perfusion and are particularly vulnerable to stenosis and occlusion in coronary artery disease. On the other hand, it also shows that uremic cardiomyopathy in the circumferential direction of myocardial damage first affects the endocardial and middle layers, while damage to the subepicardial myocardium occurs at the end, longitudinal walking endocardial layer of the myocardium is responsible for the longitudinal movement of the heart, close to the chambers of the heart, is rich in Purkinje fibers, and extremely sensitive to ischemia, when the myocardium is ischemic, it will lead to microvascular dysfunction and myocardial fibrosis, which affects the cardiac muscle movement [33], the circumferentially traveling middle myocardium, on the other hand, is the thickest part of the left ventricular wall and bears the main contractile function and is responsible for the circumferential motion of the left ventricle. When the subendocardial myocardium is in a contractile state, the subepicardial myocardium is in a relatively quiescent state and is subjected to less stress, so myocardial damage occurs later [34]. It also illustrates that uremia-induced myocardial damage does not occur simultaneously in all three layers of myocardium at different levels and is differentially affected by various factors. Comparative analysis of global CS across ventricular segments revealed significantly higher values at the apical level compared to both basal and mid-ventricular segments, and the overall contraction direction of the left ventricle is from the apex toward the basal segment [35]. The basal ventricular segment demonstrates particular sensitivity to afterload due to its anatomical proximity to the aortic valve, in contrast to mid-ventricular and apical segments. This physiological characteristic renders the basal segment especially vulnerable in uremic patients, who frequently present with comorbid hypertension and aortic valve sclerosis – conditions that substantially increase mechanical stress on this region. Alternatively, the observed strain pattern may reflect inherent heterogeneity in ventricular wall stress distribution. Biomechanical studies have established that left ventricular wall stress exhibits an apex-to-base gradient, with progressive augmentation toward the basal segments. This gradient correlates inversely with myocardial strain, as demonstrated by prior investigations showing a linear negative relationship between these parameters [36].

Based on this the present study was conducted to analyze the correlation between stratified strain parameters and Hb in the total population of the three groups, and the results showed that there was a correlation between the levels of Endo-2CS, Myo-2CS, Epi-2CS and Hb, and there was a correlation between the levels of Endo-3CS, Myo-3CS, Epi-3CS and Hb, which indicated that myocardial strain decreased with the decrease of Hb. Analyzing the reason for this may be that renal failure in uremic patients led to a decrease in erythropoietin (EPO), which affected the hematopoietic function of the bone marrow and led to a decrease in Hb, or it may be that the uremic patients with obstacles to toxin discharge accelerated erythrocyte apoptosis, and the lower Hb in the LVH group compared with that in the LVN group, the reason for this may be that anemia increases the burden on the heart, leading to myocardial hypertrophy, while decreased cardiac function leads to insufficient renal perfusion, further aggravating anemia. Therefore, the LVH group suffered more severe damage.

This study employed ROC curve analysis to evaluate layered strain parameters for predicting LV systolic dysfunction in patients with LVH. Our results demonstrated that Endo-2CS, Myo-2CS, Epi-2CS, Interaction-2CS, Endo-3CS, Myo-3CS and Epi-12CS effectively identified LV systolic dysfunction. Among these parameters, Interaction-2CS showed the highest predictive accuracy with an AUC of 0.749. Although Epi-3CS correlated with both Endo-3CS and Myo-3CS, it did not significantly predict LV myocardial motion abnormalities in LVH patients. To evaluate the independent effect of LVH on myocardial strain, we further adjusted for the confounding factor of Hb. The analysis results confirmed that LVH is an independent determinant of impaired circumferential strain. This indicates that, in uremic patients, the detrimental effect of LVH on myocardial systolic function outweighs the contribution of anemia, underscoring the importance of preventing and reversing LVH itself.

## Limitations

However, this study has several limitations. First, the relatively small sample size may affect the statistical power of our findings, warranting future studies with larger cohorts to confirm our results. Second, as a single-center investigation, the potential for selection bias exists, which may limit the external validity and generalizability of our conclusions. In addition, this study did not collect or analyze certain biochemical markers of myocardial injury, such as natriuretic peptides, brain natriuretic peptides, and troponin. Furthermore, notwithstanding our standardization of measurement timing to the second post-dialysis day, fluid status is not static during the interdialytic period. This persistent variation remains a potential source of confounding for measurements of left ventricular dimensions and strain. Finally, while the myocardium exhibits three-dimensional motion, the VVI stratified strain technique is limited to two-dimensional planar analysis. This spatial dependence restricts our ability to comprehensively assess the motion characteristics of each segment of the myocardium. All the limitations mentioned above represent important directions that need to be addressed and improved in our future research.

## Conclusion

In conclusion, our study demonstrated that myocardial damage in the basal segment occurs in uremic patients even when LVEF is normal. Furthermore, in uremic patients with LVH, this damage is more pronounced, particularly in the anterior septum. The VVI stratified strain technique offers an early and quantitative assessment of cardiac function in patients with LVH, allowing for sensitive and accurate evaluation of left ventricular myocardium.

## Supporting information

S1 Data Complete dataset for all study participants, including demographic, laboratory indices, and left ventricular global/layer-specific strain parameters. (XLS)
